# Cardiac Function in Women with and Without Previous Assisted Reproductive Technology: A Prospective Observational Cohort Study

**DOI:** 10.3390/jcm14155366

**Published:** 2025-07-29

**Authors:** Freya Baird, Eleni Kakouri, Iulia Huluta, Ippokratis Sarris, Sesh K. Sunkara, Kypros H. Nicolaides, Nick Kametas

**Affiliations:** 1 King’s Fertility, 1st Floor, Fetal Medicine Research Institute, 16–20 Windsor Walk, Denmark Hill, London SE5 8BB, UK; ippokratis.sarris@kingsfertility.co.uk (I.S.); sesh.sunkara@kcl.ac.uk (S.K.S.); 2 Department of Women’s Health, Faculty of Life Sciences and Medicine, King’s College London, Great Maze Pond, London SE1 9RT, UK; kypros@fetalmedicine.com (K.H.N.); nick.kametas@kcl.ac.uk (N.K.); 3 Fetal Medicine Research Institute, 16–20 Windsor Walk, Denmark Hill, London SE5 8BB, UK; eleni.kakouri@nhs.net (E.K.); iuliahuluta16@gmail.com (I.H.)

**Keywords:** in-vitro fertilization, frozen embryo transfer, maternal circulation, echocardiography, cardiovascular disease

## Abstract

**Background**: Previous research has linked hypertensive disorders of pregnancy (HDP) and long-term cardiovascular disease (CVD) with assisted reproductive technology (ART). It is not clear whether this reflects the background population cardiovascular profiles or whether ART independently increases the long-term risk for CVD and alters cardiovascular function. Furthermore, CVD has been associated with pathological cardiovascular function before and after the establishment of the disease. The aim of this study was to compare cardiac function in women attending for ART between those who had previous treatment and those who had not after controlling for demographic characteristics which have been shown to affect cardiovascular function. **Methods**: This was a prospective observational cohort study at a London fertility clinic. Women were consecutively enrolled between May 2021 and March 2022. Maternal demographics and cardiac function using transthoracic echocardiography were assessed before the current treatment cycle in the mid-luteal phase of the menstrual cycle. Maternal demographics included age, body mass index, smoking, race, and parity. Cardiovascular parameters included blood pressure and indices of left-ventricular systolic and diastolic function. Differences between cardiac variables after controlling for maternal demographics and history of previous ART were assessed by multivariate linear regression. **Results**: There were 232 healthy women who agreed to participate in the study; of those, 153 (58%) had undergone previous ART. After controlling for maternal demographic characteristics, previous assisted reproductive technology was not an independent predictor of cardiac function. **Conclusions**: Previous ART is not associated with significant changes in cardiac function.

## 1. Introduction

Worldwide, it is estimated that over 10 million children have been born as a result of assisted reproductive technology (ART) [1], with over 2.5 million cycles performed every year [2]. There is a reported association between ART and pregnancy-related complications, including hypertensive disorders of pregnancy (HDP) [3], fetal growth restriction (FGR) and pre-term birth (PTB) [4]. These complications are also recognized as sex-specific risk factors for cardiovascular disease (CVD) [5,6,7], with premature age of menopause and adverse pregnancy outcomes now codified as risk-enhancing factors for atherosclerotic CVD in cardiovascular and obstetric society guidelines [8].

It is known that women with infertility are more likely to have established risk for CVD, including obesity, hypertension, and diabetes. For example, subfertility has been linked with metabolic syndrome, which occurs in 30–40% of women with polycystic ovary syndrome (PCOS) [9,10]. It is not clear whether this increased risk for CVD reflects the background population cardiovascular profile in those with subfertility or whether ART independently increases the long-term risk.

Limited research has studied ART and its link to CVD, with studies providing heterogeneous results. A recent systematic review and meta-analysis reported on potential links between fertility therapy and subsequent cardiovascular outcomes [11]. Whilst data pooled from two studies found an increased risk of stroke in women receiving ART compared to those who did not [12,13], there was conflicting data on the risk of fertility therapy and the risk of hypertension, with two studies suggesting a protective effect [12,14] and one study reporting a potentially harmful effect [13].

CVD has been associated with pathological cardiovascular function before and after the establishment of the disease. Echocardiography provides significant prognostic information both for prevention and detection of early disease in asymptomatic populations. [15,16]. Studies investigating women undergoing fresh IVF cycles have demonstrated increased cardiac output (CO) and reduced peripheral vascular resistance (PVR) at the peak of oestradiol levels that do not persist at the time of embryo transfer [17]. However, there are no studies investigating the long-term effect of ART treatment on cardiac function. In this study, we performed echocardiography in women attending for ART and compared cardiac function between women who had previous treatment and those who did not. We aimed to assess if previous ART negatively impacts cardiac function independently from demographic characteristics known to increase CVD risk.

## 2. Materials and Methods

### 2.1. Study Population and Demographic Characteristics

This was a prospective observational cohort study carried out at King’s Fertility, London, UK, in collaboration with the Fetal Medicine Research Institute, London, UK. Ethics approval was granted by the local ethics committee (Wales REC 7: 20/WA/03233). Patients deemed eligible to participate included women over 18 years of age who were scheduled to start their ART treatment cycle. The treatment cycles included both fresh IVF/ICSI cycles and frozen embryo transfer (FET) cycles (both natural or natural modified and medicated FET protocols). Women attending for their first visit for a mid-luteal phase, baseline transvaginal ultrasound scan, prior to the commencement of their planned treatment cycle were approached. Enrolment was carried out between May 2021 and March 2022, and written consent was obtained from patients who agreed to participate. Exclusion criteria were cardiac, renal, or liver disease and diabetes. Participants’ electronic patient records, stored on the IDEAS software package, Version 7, (Mellowood Medical Inc., Toronto, ON, Cananda), were reviewed to establish background medical history and to ascertain previous fertility treatment history. Patients were categorized as having had previous ART treatment if they had completed at least one cycle of IVF/ICSI with or without a fresh embryo transfer. Ovulation induction and intra-uterine insemination cycles were not counted in this category.

Maternal demographics and baseline cardiovascular status were assessed at presentation to the clinic. The demographic details recorded included age, height and weight, self-reported racial origin (White, Black, South Asian, East Asian, and Other), smoking status (yes/no), and parity. The maternal weight and height were measured, and these were used to calculate body mass index (BMI) in kg/cm^2^ and body surface area (BSA) according to the following formula: 0.007184 × W0.425 × H0.725 [18].

### 2.2. Fertility Parameters

Anti-mullerian hormone (AMH) levels (expressed as pmol/L) were measured by an autoanalyzer utilizing an enzymatically amplified two-site immunoassay (Roche Elecsys, Mannheim, Germany) The endometrial thickness (ET) and antral follicle count (AFC) were obtained via transvaginal 2-dimensional ultrasound at the time of the study using a 3.8–9.3 MHz transvaginal transducer (Voluson E10 ultrasound machine, GE Healthcare, Chicago, IL, USA). The AFC was assessed in accordance with consensus opinion by the International Society of Ultrasound in Obstetrics and Gynaecology [19].

### 2.3. Blood Pressure and Transthoracic Echocardiography

Blood pressure (BP) was assessed using a mercury sphygmomanometer (Accoson Dekamet, AC Cossor & Son (Surgical) Ltd., London, UK) in accordance with the guidelines established by the British Hypertension Society [20]. Mean arterial pressure (MAP) was derived from the standard formula: MAP = (BP systolic + (2 × BP diastolic))/3.

Cardiac assessment included 2D, M-mode, power wave and colour Doppler and speckle tracking echocardiography (STE) using a 3.5 MHz transducer (Toshiba Aplio CV, Toshiba Corporation, Tokyo, Japan) according to the American Society of Echocardiography guidelines [21].

Using the cross-sectional area of the left-ventricular (LV) outflow tract and the velocity time integral of the pulsed Doppler subaortic waveform, which was recorded in the five-chamber view, stroke volume (SV) was calculated. Following this, cardiac output could be determined by multiplying the heart rate with stroke volume. The following equation was then used to calculate peripheral vascular resistance (PVR): MAP × 80/cardiac output.

Two-dimensional guided M-mode was used to assess LV long axis function using the apical four-chamber view with the septal and lateral sides of the mitral valve annulus. The apical four-chamber view was used, in addition, to assess LV filling dynamics and thereby evaluate diastolic function. Transmitral flow was determined with the sample volume positioned level with the tips of the mitral leaflets when in diastole in the fully open position. The peak E:A ratio was calculated from the peak velocity of late atrial (A) and early atrial (E) filling.

We determined the mitral closing-to-opening time (a) at LV ejection time (b) from start to end of the Doppler subaortic waveform pattern. We then measured the period between closing and opening of the mitral valve. The Tei index was calculated as (a − b)/b.

Doppler tissue imaging was performed using a 3.5 mm sample volume at the septal aspect and 5 mm volume for the lateral aspect of the mitral annulus acquired in the four-chamber view [22]. The peak velocity of early (E′) and late (A′) diastolic filling and peak systolic velocity (S’) were obtained. Both isovolumetric relaxation and contraction times (IVRT/IVCT) were assessed at both septal and lateral sites. The transmitral E:E′ ratio was determined for the septal and lateral margins of the mitral annulus, which has been demonstrated to reflect both pulmonary capillary wedge pressure and left-atrial pressure.

All cardiac assessments were performed by five experienced sonographers trained to perform cardiac echo sonography.

### 2.4. Statistical Analysis

The normality of the distribution of the data was assessed by the Kolmogoroff–Smirnoff test. Differences in demographics and study parameters between women with history of previous ART and those without were compared with the chi-squared test for categorical data and Student’s *t*-test for normally distributed and the Kruskal–Wallis test for non-normally distributed data. Backward stepwise multivariate regression analysis was used to examine the independent effect of maternal characteristics and previous history of ART on fertility and cardiac parameters.

The sample size for the two groups (women with and without a history of previous ART) was based on the study of Manau et al. [17], who demonstrated an increase in cardiac output of 0.2 L/min in women undergoing fresh IVF cycles. Assuming that this difference would be sustained post the IVF cycle, for a Type 1 error (a) of 0.01 and a Type 2 error (b) of 0.01 (i.e., power of 95%), 60 women with and 30 women without previous ART treatment needed to be recruited.

The statistical software package SPSS (released 2010, IBM SPSS Statistics for Windows, Version 29.0, IBM Corporation, Armonk, NY, USA) was used for data analysis, with *p* < 0.05 deemed to be statistically significant.

## 3. Results

**Figure 1:**
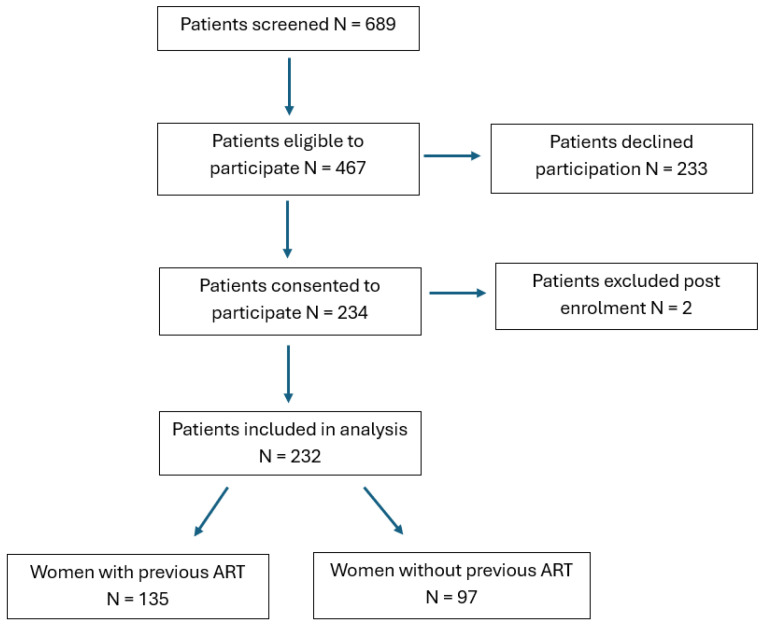
Flow chart demonstrating patient recruitment pathway.

In total, 232 women were eligible and included in the final analysis, including 135 (58%) women who had previously undergone ART and 97 (42%) who were treatment-naïve. There were no patients with cardiac, renal, or liver disease or diabetes who were approached, and therefore, none needed to be excluded. Details of the recruitment process are summarized in Figure 1.

Previous treatment included fresh IVF and frozen embryo transfer cycles with and without resultant pregnancies. A summary of maternal demographics for the whole cohort and a comparison between the two groups are presented in Table 1. In the group with previous ART, compared to women in their first treatment cycle, there was a higher median age, there was a higher incidence of nulliparous women and Black and South Asian women, but there was no significant difference between groups in BMI, BSA, or smoking status. Similarly, there were no statistically significant differences in the fertility and cardiac function parameters between the two groups, as presented in Table 2, apart from a small difference in biplane LV ejection fraction, which was lower in the group who had previous ART treatment compared to no treatment.

The results of the multivariate regression analysis are presented in Table 3 and Table 4, with the former showing the statistical significance of each variable when they are all included in the model and the latter the regression coefficients of the final model.

Regarding fertility parameters, independent predictors for AMH were age (*p* = 0.004) and BMI (Table 3, *p* = 0.01), and for AFC, it was age (Table 3, *p* = <0.001). However, no demographic parameter was associated with endometrial thickness. These demographics in relation to AMH and AFC were still found to be statistically significant in the final model (Table 4).

When assessing the cardiac variables against all maternal demographics (Table 3), with the exception of the biplane LV ejection fraction, mitral valve E-wave maximum, and the average global longitudinal strain, where race was found to be statistically significant, (*p* = 0.02), (*p* = 0.04), and (*p* = 0.004), respectively, the predominant demographic of significance was BSA. Within the subgroup of haemodynamic variables, BSA was found to be statistically significant for mean arterial pressure (*p* = 0.001), LV outflow tract (<0.001), LV outflow tract velocity peak (*p* = 0.02), LV velocity time integral (*p* = 0.005), LV stroke volume (<0.001), LV cardiac output (<0.001), and peripheral vascular resistance (0.001). This significance persisted in the final model of the multivariate regression analysis for all above-mentioned variables (Table 4). For the remaining cardiac variables, again, BSA was the predominant significant demographic: biplane LV end-diastolic volume (*p* = <0.001), biplane LV end-systolic volume (*p* = <0.001), left-atrial area (*p* = <0.001), left-atrial volume (*p* = <0.001), mitral valve a-lateral (*p* = 0.004), mitral valve s-septal (0.02), LV end-diastolic diameter (*p* = <0.001), LV end-systolic diameter (*p* = <0.001), LV mass (*p* = <0.001), and average global longitudinal strain (*p* = <0.001) (Table 3). Again, this significance persisted in the final model of the multivariate regression analysis (Table 4).

**Figure 2:**
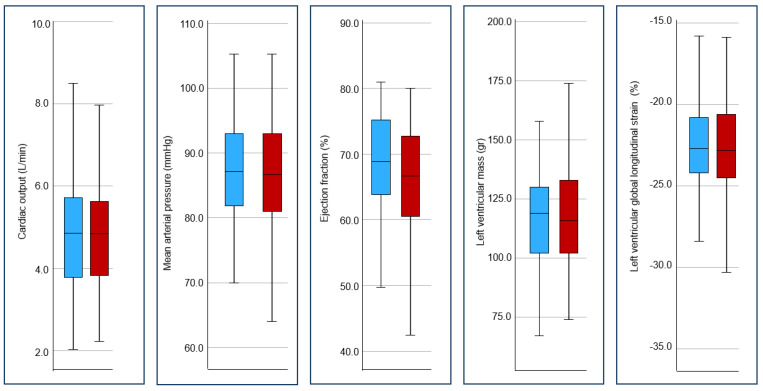
Comparison between no previous ART (blue) and previous ART (red) for cardiac variables.

Following adjustment for other maternal characteristics, there was no independent contribution from the history of ART in any cardiac variables on the multivariate regression analysis (Table 4). The effect of ART on CO, MAP, EF, LV mass, and LV global longitudinal strain is presented in Figure 2.

## 4. Discussion

This is the first study to investigate cardiac function in women who have had previous ART treatment compared to those who are treatment-naïve. We examined healthy women in whom common cardiovascular risks, such as age, ethnicity, smoking and BMI/BSA, were adjusted for in the final analysis and found that previous ART was not an independent predictor of cardiac function.

Standard echocardiography in patients without a history of CVD has been reviewed in a meta-analysis and has shown to predict fatal and non-fatal cardiovascular events and all-cause mortality [15]. In addition, the investigations performed in our study included STE, which was used to evaluate left-ventricular strain. Whilst this modality is prognostic in patients with established CVD, STE, particularly global longitudinal strain, has been shown to detect subclinical dysfunction before any changes are identified in ejection fraction, offering further prognostic value in identifying risk for CVD [23].

Strengths of the study include its prospective design and the standardized protocol for measurement of cardiovascular parameters. In addition, all cardiac assessments were performed by experienced sonographers trained to perform cardiac sonography, thereby minimizing a potential confounding variable when measuring the parameters of interest. A limitation of the study was that as there is no data presenting cardiac changes in frozen embryo transfer cycles; therefore, our power calculation was based on data for women in fresh IVF cycles only. Additionally, as women were voluntarily taking part in the study, it is possible that those recruited in this group might be confounded by selection bias (such as volunteer bias). This might affect our study’s external validity. In addition, whilst common modifiable and non-modifiable risk factors for CVD were accounted for in this study, we note that there are further factors that were not evaluated and corrected for, including hypercholesterolemia, diet and alcohol intake, and family history, which should be considered when reviewing the results presented.

Heterogenous results have been shown in previous research in this area. A recent systematic review investigated the acute changes in maternal haemodynamics associated with IVF treatment [24]. However, the included studies reported on fresh cycles only and predominantly used a long down-regulation (agonist) protocol. Assessment of cardiac function was reported in five studies within the review, all with limited numbers and with varying results. Findings included a significant increase in left-ventricular dimension at both end-diastole and end-systole and a significant increase in cardiac output [17,25]. A transient increase in left-ventricular end-diastolic volume was reported in one study along with a decrease in left-ventricular ejection fraction, with both trending towards baseline after the peak estradiol level was recorded [26]. Furthermore, one study indicated changes in the modulation of heart rate variability [27]. A significant change in biventricular systolic and diastolic function was not demonstrated [28]. It was noted by the reviewers that due to the heterogeneity of the included studies, the meta-analysis was limited. Therefore, the variability in outcomes and the small sample size mean no specific conclusions could be drawn.

Whilst we found no association between previous ART treatment and subsequent cardiovascular changes when corrected for background cardiac risk factors, both BSA and age were found to independently affect cardiovascular parameters as expected. Although multiple studies have highlighted the link with age [29] and BSA [30,31] on cardiovascular risk, confirming these changes in women seeking fertility treatment highlights to the clinician the importance of optimizing cardiovascular health prior to ART treatment. This is not only to reduce the risk of complications within pregnancy but also for the long-term cardiovascular health of the patient.

While no independent associations between ART and cardiovascular function were detected in this study, larger cohort studies exploring both short-term reversible and longer-term changes in cardiovascular function in treatment, pregnancy, and beyond, adjusting further for other identifiable risk factors for CVD, including hypercholesterolemia, may inform on the future risk of cardiovascular disorders during gestation and later life.

## Figures and Tables

**Table 1:** Demographic characteristics in the whole cohort and in the two groups of women with and without previous history of ART.

Variable	Total Population (*n* = 232)	Women with Previous ART (*n* = 135)	Women Without Previous ART (*n* = 97)	*p*-Value
**Demographic details**				
Age (years)	37.0 (34.0–40.0)	38.0 (35.0–40.0)	36.0 (33.5–39.0)	0.01
Height (cm)	166.0 (161.0–170.0)	165.0 (160.0–169.5)	166.0 (161.0–170.00)	0.69
Weight (kg)	66.3 (60.0–73.1)	67.0 (59.8–74.8)	65.3 (60.0–72.4)	0.78
Body Mass Index (kg/m^2^)	24.2 (21.8–27.4)	24.1 (21.3–28.3)	24.3 (22.0–26.3)	0.66
Body Surface Area (m^2^)	1.7 (1.6–1.8)	1.7 (1.6–1.8)	1.7 (1.6–1.8)	0.80
Race				0.03
White, *n* (%)	194 (83.6)	104 (77.0)	90 (92.8)	
Black, *n* (%)	14 (6.0)	11 (8.1)	3 (3.1)	
South Asian, *n* (%)	15 (6.5)	13 (9.6)	2 (2.1)	
East Asian, *n* (%)	3 (1.3)	2 (1.5)	1 (1.0)	
Other, *n* (%)	6 (2.6)	5 (3.7)	1 (1.0)	
Smoking, *n* (%)	3 (1.3)	1 (0.7)	2 (2.1)	0.38
Nulliparous, *n* (%)	178 (76.7)	91 (67.4)	87 (89.7)	<0.001

ART: assisted reproductive technology.

**Table 2:** ART and cardiac function variables in the whole cohort and in the two groups of women with and without previous history of ART.

Variable	Total Population (*n* = 232)	Women with Previous ART (*n* = 135)	Women Without Previous ART (*n* = 97)	*p*-Value
**ART variables**				
Anti-Müllerian hormone (pmol/L)	12.3 (6.9–19.9)	12.4 (7.3–20.6)	12.1 (6.4–18.2)	0.53
Endometrial thickness (mm)	9.3 (7.7–11.1)	9.2 (7.5–10.7)	9.9 (8.0–113)	0.07
Antral follicle count	13.0 (8.0–20.0)	13.0 (8.0–20.5)	14.0 (8.0–19.0)	0.77
**Cardiac function variables**				
**Haemodynamic variables**				
Mean arterial pressure (mmHg)	86.8 (81.0–93.0)	86.7 (81.0–93.1)	87.0 (81.1–93.0)	0.61
Left-ventricular outflow tract (mm)	19.2 (18.5–20.5)	19.4 (18.6–20.7)	19.1 (18.5–20.4)	0.40
LV outflow tract velocity peak (cm/s)	111.2 (97.7–124.6)	109.1 (96.4–120.4)	110.3 (96.3–128.0)	0.20
LV velocity time integral (cm/s)	25.0 (21.7–27.6)	24.8 (21.5–27.0)	24.2 (20.9–28.0)	0.80
LV stroke volume (mL)	71.6 (59.3–89.5)	71.5 (60.9–89.2)	71.5 (57.3– 89.5)	0.58
Heart rate (b/min)	66.0 (59.0–74.0)	65.0 (59.0–72.0)	64.0 (59.0–74.0)	0.70
LV cardiac output (L/min)	4.9 (3.9–5.7)	4.8 (3.8–5.6)	4.9 (3.8–5.7)	0.82
Peripheral vascular resistance (dynes/s/cm^−5^)	1497.6 (1216.6–1780.7)	1472.3 (1201.2–1754.2)	1500.0 (1220.4–1859.8)	0.52
**Left-ventricular systolic function**				
Biplane LV end-diastolic volume (mL)	72.6 (63.4–81.6)	72.3 (64.3–79.8)	72.6 (62.6–82.3)	0.83
Biplane LV end-systolic volume (mL)	26.6 (23.2–31.1)	27.3 (23.6–30.8)	25.9 (22.8–32.0)	0.19
Biplane LV ejection fraction (%)	63.8 (58.6–68.7)	62.7 (58.0–67.7)	64.6 (61.6–70.3)	0.04
LV Isovolumic contraction time (ms)	58.0 (53.0–68.5)	58.0 (51.5–69.0)	58.0 (53.0–67.0)	0.96
LV ejection time (ms)	294.0 (278.0–311.0)	297.0 (281.0–312.5)	292.0 (273.5–308.0)	0.06
LV myocardial performance index	0.4 (0.3–0.5)	0.4 (0.3–0.5)	0.4 (0.3–0.5)	0.25
Left-atrial area (cm^2^)	11.7 (9.9–12.9)	11.1 (9.5–12.8)	11.9 (10.4–13.2)	0.09
Left-atrial volume (mL)	27.5 (21.8–33.2)	26.6 (20.4–32.9)	28.6 (24.3–33.0)	0.11
**Left-ventricular diastolic function**				
Mitral valve E-wave maximum velocity (cm/s)	82.3 (72.3–92.2)	80.1 (70.7–90.0)	82.8 (72.8–93.9)	0.24
Mitral valve A-wave maximum velocity (cm/s)	44.9 (33.8–54.4)	44.9 (33.8–54.4)	44.6 (32.5–54.7)	0.87
Mitral valve E/A-wave ratio	1.8 (1.5–2.3)	1.8 (1.5–2.3)	1.8 (1.5–2.4)	0.55
Mitral valve e-lateral (cm/s)	15.0 (12.4–17.3)	14.9 (12.3–16.8)	14.9 (12.6–17.6)	0.41
Mitral valve a-lateral (cm/s)	9.4 (7.6–11.8)	9.5 (7.6–11.9)	9.2 (7.6–11.5)	0.79
Mitral valve s-lateral (cm/s)	9.4 (8.0–11.7)	9.5 (7.9–11.5)	9.4 (8.4–11.6)	0.74
Mitral valve e-septal (cm/s)	11.3 (10.0–13.3)	11.3 (9.7–13.4)	11.5 (10.3–13.1)	0.35
Mitral valve a-septal (cm/s)	9.0 (7.9–10.7)	9.0 (7.6–10.5)	9.2 (8.2–10.9)	0.27
Mitral valve s-septal (cm/s)	9.2 (7.9–10.4)	9.3 (8.2–10.3)	9.3 (7.8–10.7)	0.71
Isovolumic relaxation time (ms)	67.0 (56.0–78.0)	64.0 (53.0–76.5)	67.0 (56.0–79.5)	0.32
**Left-ventricular m-mode**				
LV intraventricular septum in diastole (mm)	10.2 (9.0–11.4)	10.2 (9.0–11.8)	10.1 (8.4–11.3)	0.21
LV end-diastolic diameter (mm)	41.5 (38.4–45.2)	41.8 (38.8–44.1)	41.3 (38.2–45.9)	0.41
LV posterior wall in diastole (mm)	9.5 (8.4–10.9)	9.2 (8.2–10.5)	9.9 (8.4–11.1)	0.13
LV end-systolic diameter (mm)	26.0 (23.2–28.5)	26.2 (23.8–28.6)	25.7 (22.7–28.5)	0.16
LV mass (g)	117.5 (102.2–132.8)	116.0 (102.0–133.2)	119.0 (103.0–130.0)	0.82
**Global and Circumferential strain**				
Average global longitudinal strain (%)	−22.7 (−24.2–20.6)	−22.8 (24.3–20.6)	−22.7 (−24.2–20.8)	0.94
Circumferential strain at the level of the mitral valve (%)	−26.1 (−31.5–20.1)	−26.9 (−31.0–20.3)	−24.4 (−31.3–19.8)	0.40
Circumferential strain at the level of the pectinate muscles (%)	−26.1 (−31.5–20.0)	−26.0 (−31.3–21.5)	−25.9 (−31.3–19.3)	0.89

ART: assisted reproductive technology, LV: left-ventricular.

**Table 3:** Multivariable regression analysis assessing the independent prediction of ART and cardiac variables with a history of previous ART after controlling for maternal characteristics. The *p*-value for each variable and the *p*-value and R-squared for the overall model are presented.

Variable	Age	Body Mass Index	Body Surface Area	Race	Smoking	Nulliparous	Previous ART	Model *p*-Value	Model R-Squared
**ART variables**									
Anti-Müllerian hormone (pmol/L)	0.004	0.01	-	0.49	0.31	0.37	0.59	0.01	0.08
Endometrial thickness (mm)	0.11	0.77	-	0.94	0.10	0.07	0.13	0.21	0.05
Antral follicle count	<0.001	0.16	-	0.21	0.22	0.35	0.08	<0.001	0.13
**Cardiac function variables**									
**Haemodynamic variables**									
Mean arterial pressure (mmHg)	0.95	-	0.001	0.97	0.99	0.97	0.53	0.13	0.06
Left-ventricular outflow tract (mm)	0.75	-	<0.001	0.77	0.76	0.53	0.18	0.05	0.07
LV outflow tract velocity peak (cm/s)	0.70	-	0.02	0.11	0.78	0.32	0.41	0.02	0.08
LV velocity time integral (cm/s)	0.87	-	0.005	0.20	0.56	0.22	0.88	0.02	0.08
LV stroke volume (mL)	0.69	-	<0.001	0.76	0.52	0.21	0.39	<0.001	0.12
Heart rate (b/min)	0.39	-	0.95	0.14	0.07	0.82	0.32	0.22	0.05
LV cardiac output (L/min)	0.40	-	<0.001	0.66	0.69	0.23	0.66	<0.001	0.12
Peripheral vascular resistance (dynes/s/cm^−5^)	0.33	-	0.001	0.80	0.95	0.12	0.26	0.03	0.08
**Left-ventricular systolic function**									
Biplane LV end-diastolic volume (mL)	0.09	-	<0.001	0.19	0.94	0.60	0.69	<0.001	0.18
Biplane LV end-systolic volume (mL)	0.05	-	<0.001	0.62	0.96	0.17	0.13	0.001	0.12
Biplane LV ejection fraction (%)	0.04	-	0.55	0.02	0.72	0.14	0.04	0.007	0.10
LV Isovolumic contraction time (ms)	0.42	-	0.51	0.13	0.59	0.98	0.91	0.42	0.04
LV ejection time (ms)	0.02	-	0.30	0.57	0.49	0.87	0.22	0.15	0.06
LV myocardial performance index	0.21	-	0.69	0.88	0.56	0.89	0.39	0.86	0.02
Left-atrial area (cm^2^)	0.51	-	<0.001	0.48	0.55	0.31	0.12	0.002	0.11
Left-atrial volume (mL)	0.54	-	<0.001	0.89	0.69	0.25	0.14	0.02	0.08
**Left-ventricular diastolic function**									
Mitral valve E-wave maximum velocity (cm/s)	0.49	-	0.24	0.04	0.68	0.78	0.34	0.17	0.05
Mitral valve A-wave maximum velocity (cm/s)	0.26	-	0.32	0.22	0.99	0.67	0.41	0.56	0.03
Mitral valve E/A-wave ratio	0.09	-	0.10	0.15	0.66	0.87	0.79	0.21	0.05
Mitral valve e-lateral (cm/s)	0.23	-	0.11	0.18	0.07	0.46	0.98	0.07	0.07
Mitral valve a-lateral (cm/s)	0.48	-	0.004	0.74	0.43	0.90	0.69	0.16	0.06
Mitral valve s-lateral (cm/s)	0.19	-	0.42	0.74	0.85	0.92	0.91	0.87	0.02
Mitral valve e-septal (cm/s)	0.08	-	0.28	0.53	0.45	0.91	0.92	0.53	0.04
Mitral valve a-septal (cm/s)	0.80	-	0.37	0.48	0.28	0.37	0.47	0.59	0.03
Mitral valve s-septal (cm/s)	0.31	-	0.02	0.05	0.16	0.78	0.31	0.41	0.08
Isovolumic relaxation time (ms)	0.45	-	0.90	0.61	0.95	0.64	0.47	0.85	0.02
**Left-ventricular m-mode**									
LV intraventricular septum in diastole (mm)	0.20	-	0.05	0.17	0.79	0.39	0.08	0.08	0.07
LV end-diastolic diameter (mm)	0.57	-	<0.001	0.43	0.98	0.86	0.58	<0.001	0.13
LV posterior wall in diastole (mm)	0.18	-	0.15	0.53	0.99	0.70	0.49	0.44	0.04
LV end-systolic diameter (mm)	0.68	-	<0.001	0.47	0.16	0.94	0.65	0.001	0.11
LV mass (g)	0.27	-	<0.001	0.75	0.94	0.39	0.37	<0.001	0.15
**Global and Circumferential strain**									
Average global longitudinal strain (%)	0.51	-	<0.001	0.002	0.85	0.30	0.86	<0.001	0.12
Circumferential strain at the level of the mitral valve (%)	0.13	-	0.93	0.67	0.66	0.37	0.32	0.73	0.03
Circumferential strain at the level of the pectinate muscles (%)	0.14	-	0.74	0.89	0.80	0.11	0.97	0.76	0.03

ART: assisted reproductive technology, LV: left-ventricular.

**Table 4:** Multivariate regression analysis for ART and cardiac function variables. Dependent variables that were not predicted from any of the independent parameters were omitted. As there was no independent contribution from nulliparity and previous ART, these predictors do not appear on the table.

Variable	Age		Body Mass Index		Body Surface Area		Race		Smoking		Model *p*-Value	Model R-Squared
	Coef. B (SE)	*p*-Value	Coef. B (SE)	*p*-Value	Coef. B (SE)	*p*-Value	Coef. B (SE)	*p*-Value	Coef. B (SE)	*p*-Value		
**ART variables**												
Anti-Müllerian hormone (pmol/L)	−0.65 (0.22)	0.004	−0.57 (0.23)	0.02	-	-	-	-	-	-	<0.001	0.06
Antral follicle count	−0.75 (0.17)	<0.001	-	-	-	-	-	-	-	-	<0.001	0.08
**Cardiac function variables**												
Haemodynamic variables												
Mean arterial pressure (mmHg)	-	-	-	-	15.13 (4.13)	<0.001	-	-	-	-	<0.001	0.05
LV outflow tract (mm)	-	-	-	-	2.85 (0.77)	<0.001	-	-	-	-	<0.001	0.06
LV tract peak velocity (cm/s)	-	-	-	-	27.74 (9.55)	0.004	-	-	-	-	0.004	0.04
LV velocity time integral (cm/s)	-	-	-	-	7.58 (2.17)	<0.001	-	-	-	-	<0.001	0.05
LV stroke volume (mL)	-	-	-	-	45.29 (8.83)	<0.001	-	-	-	-	<0.001	0.10
LV cardiac output (L/min)	-	-	-	-	3.03 (0.62)	<0.001	-	-	-	-	<0.001	0.10
PVR (dynes/s/cm^−5^)	-	-	-	-	−778.85 (220.08)	<0.001	-	-	-	-	<0.001	0.05
**Left-ventricular systolic function**												
Biplane LV end-diastolic volume (mL)	-	-	-	-	37.66 (6.03)	<0.001	-	-	-	-	<0.001	0.15
Biplane LV end-systolic volume (mL)	−0.23 (0.11)	0.042	-	-	13.61 (3.02)	<0.001	-	-	-	-	<0.001	0.09
Biplane LV ejection fraction (%)	0.31 (0.13)	0.021	-	-	-	-	-	-	-	-	0.021	0.02
LV ejection time (ms)	1.21 (0.45)	0.008	-	-	-	-	-	-	-	-	0.008	0.03
Left-atrial area (cm^2^)	-	-	-	-	4.42 (1.03)	<0.001	-	-	-	-	<0.001	0.08
Left-atrial volume (mL)	-	-	-	-	14.84 (3.94)	<0.001	-	-	-	-	<0.001	0.06
**Left-ventricular diastolic function**												
Mitral valve e-lateral (cm/s)	-	-	-	-	-	-	-	-	−4.46 (1.98)	0.025	0.025	0.02
Mitral valve a-lateral (cm/s)	-	-	-	-	4.22 (1.37)	0.002	-	-	-	-	0.002	0.04
Mitral valve s-septal (cm/s)	-	-	-	-	2.18 (0.95)	0.022	-	-	-	-	0.022	0.02
**Left-ventricular m-mode**												
LV intraventricular septum in diastole (mm)	-	-	-	-	1.87 (0.89)	0.037	-	-	-	-	0.037	0.02
LV end-diastolic diameter (mm)	-	-	-	-	11.63 (2.18)	<0.001	-	-	-	-	<0.001	0.11
LV end-systolic diameter (mm)	-	-	-	-	8.23 (1.90)	<0.001	-	-	-	-	<0.001	0.08
LV mass (g)	-	-	-	-	64.98 (10.77)	<0.001	-	-	-	-	<0.001	0.14
**Global and Circumferential strain**												
Average global longitudinal strain (%)	-	-	-	-	5.17 (1.41)	<0.001	*	0.002	-	-	<0.001	0.12

ART: assisted reproductive technology, LV: left-ventricular, PVR: peripheral vascular resistance. * The full regression model is provided in the Results section—Table 3.

## Data Availability

The datasets presented in this article are not readily available due to restrictions from the Human Fertilisation and Embryology Authority (HFEA).
